# Rheumatoid arthritis mimicking metastatic squamous cell carcinoma

**DOI:** 10.1186/1758-3284-3-26

**Published:** 2011-05-14

**Authors:** Fernando Gomez-Rivera, Adel K El-Naggar, Nandita Guha-Thakurta, Michael E Kupferman

**Affiliations:** 1 Otorhinolaryngology - Head and Neck Surgery/Otolaryngology, The University of Houston Health Science Center, Houston, Texas, USA; 2 Department of Pathology, The University of Texas MD Anderson Cancer Center, Houston, Texas, USA; 3 Department of Radiology, The University of Texas MD Anderson Cancer Center, Houston, Texas, USA; 4 Department of Head and Neck Surgery, The University of Texas MD Anderson Cancer Center, Houston, Texas, USA

**Keywords:** Rheumatoid, arthritis, nodule, hyoid, larynx

## Abstract

We report a case of a cervical rheumatoid nodule in close relation to the hyoid bone mimicking a metastatic carcinoma. A 74-year-old female with a 15-year history of rheumatoid arthritis (RA) on treatment with methotrexate presented with tenderness of the right base of tongue. Imaging demonstrated a 1.4 cm cystic lesion at the hyoid bone. Biopsies were unsuccessful and the patient required surgical resection of the mass. A trans-cervical approach was used. Pathology revealed a necrotizing granuloma compatible with rheumatoid etiology. The clinician should be aware that, in a patient with a neck mass, in the presence of active RA, rheumatoid nodules should be part of the differential diagnosis.

## Introduction

Rheumatoid nodules have been reported at unusual sites, including the eyelid, soles, the spine and various viscera. RA also can affect the larynx, ear and nose with various otorhinolaryngological manifestations.

The incidence of laryngeal involvement in RA ranges from 13 to 75%[1]. RA typically involves the cricoarytenoid joint (CJ), but rheumatoid nodules can occur within the vocal cords[2]. Under approval of the MD Anderson Cancer Center Institutional Review Board, we report a case of a cervical rheumatoid nodule in close relation to the hyoid bone mimicking a metastatic carcinoma.

## Case

A 74-year-old female initially noted tenderness of the right base of tongue and right aural fullness one year prior to presentation. No additional head and neck symptoms were elicited. The patient had rheumatoid arthritis, for which she was currently being treated with methotrexate. An MRI of the neck was performed, which demonstrated a 1.4 cm cystic lesion at the hyoid bone protruding into the pre-epiglottic fat. This led to a provisional diagnosis of malignancy to the hyoid bone. On evaluation at our institution, the patient was noted to have tenderness to palpation at the right base of tongue, but no palpable masses. On flexible fiberoptic laryngoscopy, no lesions were noted. Normal vocal cord motion was seen bilaterally, and there was no palpable adenopathy in the neck.

**Figure 1:**
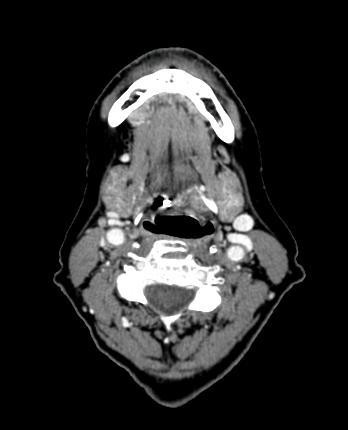
**Post contrast axial CT**. Post contrast axial CT scan demonstrating a heterogeneous, well circumscribed lesion in the left pre-epiglottic space abutting the posterior surface of the hyoid bone at the junction of the body and the cornua.

A CT scan revealed a small, well-circumscribed, ovoid mass with central necrosis in the left pre-epiglottic space, abutting the posterior surface of the hyoid bone (Figure 1). Neither an ultrasound guided fine-needle aspiration biopsy, nor a core needle biopsy, despite multiple attempts, was successful in identifying the histology.

It was decided to proceed with surgical resection of the mass. Intraoperative laryngoscopy revealed negative findings in the vallecula and in the larynx. A trans-cervical approach to the left neck was undertaken. After division of the infrahyoid musculature, the lesion was identified and circumferentially resected with the lateral cornu of the hyoid bone. Frozen section analysis was consistent with a rheumatoid nodule. The laryngeal framework was then reconstructed by reapproximating the strap muscles and hyoglossus muscle to the hyoid to ensure the stability of the larynx.

**Figure 2:**
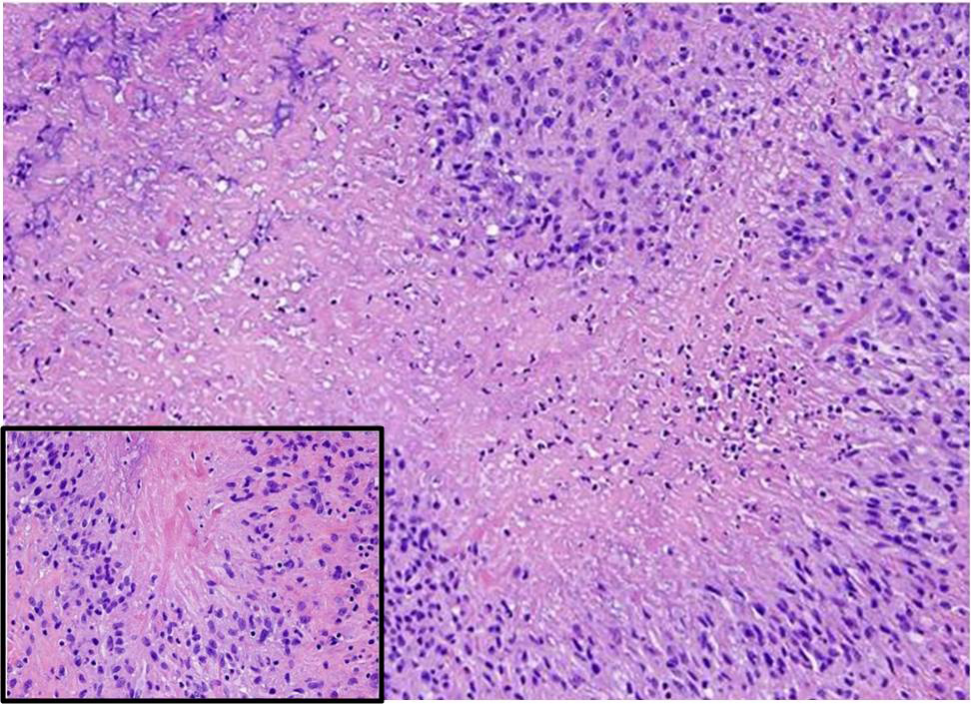
**Photomicroscopy**. The lesion composed of multiple irregular, fairly demarcated nodules with central eosinophilic necrosis (**×20**) lined by thick peripheral layer of palisading mono-nuclear cells (inset **×40**) characteristic of rheumatoid nodules.

Final pathology evaluation revealed a necrotizing granuloma, compatible with rheumatoid etiology (Figure 2). Stains for fungus and acid fast bacilli were negative. The patient was discharged from the hospital in stable condition and, at 6-month follow-up, had no significant sequelae. Her RA continued to require treatment with methotrexate.

## Discussion

We present the case of a patient with a tender mass in the pre-epiglottic region at the level of the hyoid bone, which final pathology demonstrated to be a rheumatoid nodule. Although rheumatoid nodules in the larynx have been described, focal involvement of the hyoid bone to our knowledge has not been reported in the literature.

Laryngeal abnormalities in RA can be seen on CT imaging in 50-75% of patients[3,4]. The prevalence ranges from 13 to 75% in various clinical studies, and between 45 and 88% in postmortem studies[5]. Although these findings indicate that laryngeal involvement of RA is common, clinical suspicion is necessary to recognize it.

In a patient presenting with a laryngeal mass, the otolaryngologist should be cognizant that the differential diagnosis should include a malignant process, more frequently squamous cell carcinoma. Other malignant considerations include adenoid cystic carcinoma, lymphoma and sarcomas. Among the benign lesions, laryngocele, amyloidosis, papilloma and inflammatory processes such as tuberculosis and rheumatoid nodules^2 ^should also be considered.

Most studies on laryngeal involvement of RA describe nodules at the CJ; however, nodules involving the hyoid, to our knowledge, have not been described. In our case, the nodule was traced to the posterior aspect of the hyoid bone. It is possible that the rheumatoid nodule had arisen from the union of the body to the lesser cornus of the hyoid, which is usually attached to the body of the bone by fibrous tissue, and occasionally to the greater cornua by distinct diarthrodial joints. This joint usually persists throughout life, but occasionally becomes ankylosed.

The presence of a new neck mass should always involve a thorough clinical history, examination and further evaluation, including imaging and directed biopsies, to exclude the presence of a malignancy. However, in patients with known RA and active disease, the clinician should be aware that, although rare, rheumatoid nodules should be included as one of the primary differential diagnoses of neck masses.

## List of abbreviations

CJ: cricoarytenoid joint; RA: rheumatoid arthritis

## Competing interests

The authors declare that they have no competing interests.

## Authors' contributions

FG participated in the collection of the data, review of the literature, drafting and edition of the manuscript. AE participated in the review and interpretation of the histopathology, NG participated in the interpretation of the imaging and edition of the manuscript, MK conceived the study, and participated in the writing and edition of the manuscript. All authors read and approved the final manuscript.

Fernando Gomez-Rivera, M.D. (FG), Adel K. El-Naggar, M.D., Ph.D. (AE), Nandita Guha-Thakurta, M.D. (NG), Michael E. Kupferman, M.D. (MK)

## Consent statement

Written informed consent was obtained from the patient for publication of this case report and accompanying images. A copy of the written consent is available for review by the Editor-in-Chief of this journal.
